# EXAMINING KNOWLEDGE ABOUT FIVE ALZHEIMER’S DISEASE–RELATED SERVICE AREAS AND ITS CORRELATES IN KOREAN AMERICANS

**DOI:** 10.1093/geroni/igae098.2974

**Published:** 2024-12-31

**Authors:** Michin Hong, Sang Lee

**Affiliations:** Indiana University Indianapolis, Indianapolis, Indiana, United States; San Jose State University, San Jose, California, United States

## Abstract

This study examined knowledge about Alzheimer’s disease (AD) related services and its predictors among Korean Americans (KAs). A total of 268 KAs in the Greater Washington metropolitan area participated and completed a cross-sectional survey. Knowledge about AD related services was assessed by asking how well they knew about each of the following areas: Diagnostic services, Alzheimer’s specialists, community-based services, home-based services, and institutional care, with four categories of responses from don’t know at all to know well. Multiple regression analyses were conducted for each service area with predictors including education, English proficiency, exposure to AD, sources and frequency of health-related information, and AD knowledge about treatment and management. KAs were more knowledgeable about community- and home-based services and institutional care than diagnostic services and Alzheimer’s specialist. All regression models except for diagnostic services were significant: Having more exposure to AD is related to being more knowledgeable in Alzheimer’s specialist, community- and home-based services, and institutional care; having more sources and frequency of health information is related to being more knowledgeable about Alzheimer’s specialist; and being more knowledgeable about AD management and treatment is related to having more knowledge about community- and home-based services, respectively. Our findings revealed specific areas of services more and less known to KAs, which needs to be addressed in educational outreach. Multivariate analyses identified variations in the predictors of different service areas while confirming the robust role of exposure to AD across the service areas, suggesting ways to increase knowledge about certain types of AD services.

